# An integrative network-based approach to identify novel disease genes and pathways: a case study in the context of inflammatory bowel disease

**DOI:** 10.1186/s12859-018-2251-x

**Published:** 2018-07-13

**Authors:** Ryohei Eguchi, Mohammand Bozlul Karim, Pingzhao Hu, Tetsuo Sato, Naoaki Ono, Shigehiko Kanaya, Md. Altaf-Ul-Amin

**Affiliations:** 1 Graduate School of Science and Technology & NAIST Data Science Center, Nara Institute of Science and Technology, Nara, Japan; 2 Department of Biochemistry and Medical Genetics, University of Manitoba, Winnipeg, Canada; 3 George and Fay Yee Centre for Healthcare Innovation, University of Manitoba, Winnipeg, Canada; 4 Department of Electrical and Computer Engineering, University of Manitoba, Winnipeg, Canada; 5 Department of Radiological Technology, Gunma Prefectural College of Health Sciences, Gunma, Japan

**Keywords:** Disease gene, Inflammatory bowel disease, Gene expression, Protein-protein interaction

## Abstract

**Background:**

There are different and complicated associations between genes and diseases. Finding the causal associations between genes and specific diseases is still challenging. In this work we present a method to predict novel associations of genes and pathways with inflammatory bowel disease (IBD) by integrating information of differential gene expression, protein-protein interaction and known disease genes related to IBD.

**Results:**

We downloaded IBD gene expression data from NCBI’s Gene Expression Omnibus, performed statistical analysis to determine differentially expressed genes, collected known IBD genes from DisGeNet database, which were used to construct a IBD related PPI network with HIPPIE database. We adapted our graph-based clustering algorithm DPClusO to cluster the disease PPI network. We evaluated the statistical significance of the identified clusters in the context of determining the richness of IBD genes using Fisher’s exact test and predicted novel genes related to IBD. We showed 93.8% of our predictions are correct in the context of other databases and published literatures related to IBD.

**Conclusions:**

Finding disease-causing genes is necessary for developing drugs with synergistic effect targeting many genes simultaneously. Here we present an approach to identify novel disease genes and pathways and discuss our approach in the context of IBD. The approach can be generalized to find disease-associated genes for other diseases.

**Electronic supplementary material:**

The online version of this article (10.1186/s12859-018-2251-x) contains supplementary material, which is available to authorized users.

## Background

Inflammatory bowel disease (IBD) causes chronic inflammation of some or all part of the digestive tract. There are two major subtypes of IBD: ulcerative colitis (UC) and Crohn’s disease (CD). Both types usually involve severe diarrhea, pain, fatigue and weight loss. IBD can bring severe situations and can lead to life-threatening complications. IBD is still not curable since there are no suitable drugs and targets for curing the disease.

IBD is an idiopathic, chronic and often disabling inflammatory disorders of the gastrointestinal tract characterized by dysregulated mucosal immune response. IBD can result in life threatening bleeding, sepsis and bowel obstruction. The pathogenesis of IBD is still elusive and therefore needs to be understood for developing cure for IBD. Genome-wide association studies (GWAS), have significantly advanced our understanding on the importance of genetic susceptibility in IBD. The GWAS performed to date together with a meta-analyasis of several GWAS have identified a total of 163 IBD loci [1]. These studies mainly focused on the common genetic variants (single nucleotide polymorphisms (SNPs)). These risk loci are asscciated to a handful of candidate genes which have small contributory effects in IBD.

Significant interest has been developed for inventing new methods based on integrating omics data for identifying disease causal genes. For example, network-based classification approaches have been developed to integrate gene expression and protein interaction data to predict breast cancer metastasis [2, 3], multiple sclerosis relapse and remissions [4] and autoimmune disease [5]. Other studies also identified subnetwork modules from integrating protein interaction data with GWAS signals for complex diseases [6].

During the past decade, a huge pile of biological data has been generated from various large-scale omics studies, prompting the scientific community to gain deeper insight into underlying biological mechanisms of different diseases. One of the interesting topics is to find disease-gene associations. Broadly speaking, a disease-gene association can be a connection reported in the literature, such as a genetic association (i.e., mutations in a given gene may lead to a specific disease), or inferred from other sources [7]. Similarities between disease symptomes and gene functions could be used to predict disease-causing genes by text mining [8]. The human diseasome was constructed by connecting diseases to shared disease-causing genes [9]. Understanding of disease relationships has been explored using different types of omics data such as biological pathways [10], transcriptome data [11, 12], biomedical ontologies [13, 14], and genome-wide association study (GWAS) data [14–17]. Recently, large-scale biological data have been analyzed based on networks, and network topology has been utilized to provide insights into diseases and their associations with genes [9, 18–20]. Because the interactions between bio-molecules play crucial roles in the cell, the topology of biological networks is likely to have various biological and clinical applications [21, 22].

Cellular functions rely on the coordinated actions of multiple genes, proteins, and metabolites. Therefore, organizing biological information in the context of networks is important for deep understanding of biological systems. Discovery of modules in biological networks helps isolate systems with disease related properties and reduces interactome complexity [23]. Proteins rarely act alone as their functions tend to be regulated. Many molecular processes within a cell are carried out by molecular machines that are built from a large number of protein components organized by their protein-protein interactions (PPIs). The disease proteins (the product of disease genes) are not scattered randomly in the interactome but tend to interact with each other. Because of incompleteness of disease genes and PPI data, the known disease genes usually fail to form observable modules in PPI networks. Out of 299 diseases only 20% of the respective known disease gene from some type of modules [24]. To compensate for such gaps to a certain extent, In the present work we focus on finding novel IBD associated genes and pathways by integrating IBD gene expression, PPIs, and known IBD genes by adapting the DPClusO network clustering algorithm we published previously.

## Results and Discussion

**Fig. 1:**
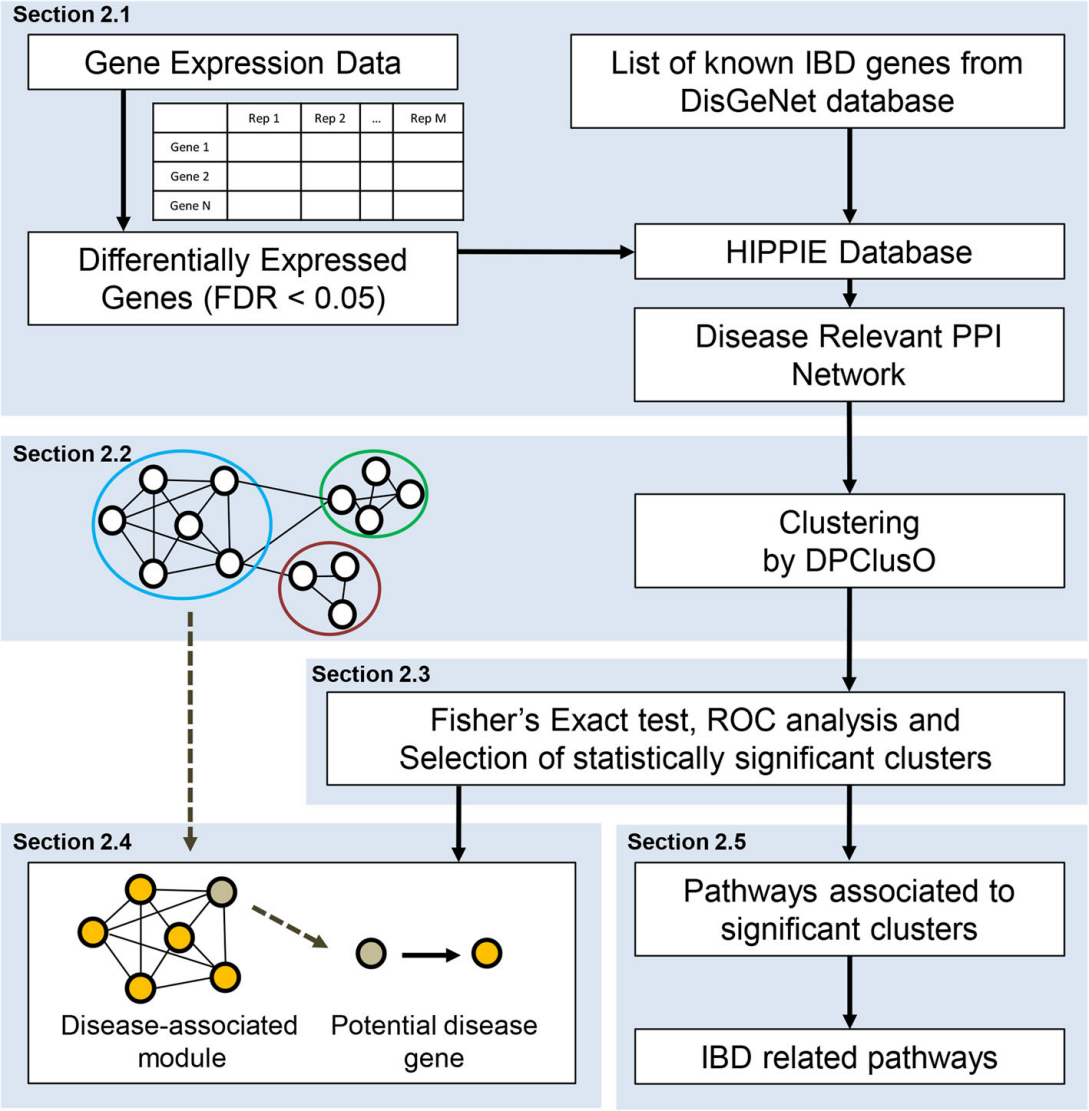
Flowchart demonstrating the major steps of the proposed approach

**Fig. 2:**
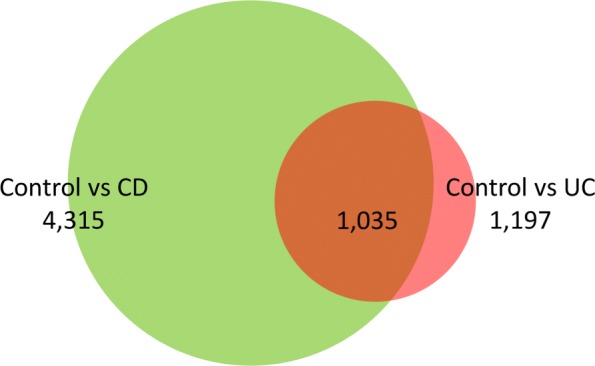
The venn diagram showing overlapping between differentially expressed genes in case of CD and UC

The method adopted in the present work has been illustrated in Fig. 1. Based on the IBD gene expression data downloaded from NCBI’s Gene Expression Omnibus (GSE57945) [25], we got 1197 and 4315 differentially expressed genes (DEGs) (with false discovery rate (FDR) < 0.05) between control and Crohn’s disease (CD) as well as control and ulcerative colitis (UC) samples, respectively. The venn diagram of the overlapping genes between these two sets is shown in Fig. 2. CD and UC are closely related diseases, hence, the differentially expressed genes are largely overlapped (1035 overlapped genes). As our focus is to find novel IBD genes and pathways by system level analysis, we took the union set of the differentially expressed genes from these two comparisons, and combined these genes to a single set consisting of 4477 genes. The differentially expressed genes are the potential candidates to be relevant to IBD.

### Construction of a disease relevant PPI network

We initially downloaded 866 genes reported in DisGeNet database [26] as IBD genes. We found that 318 of the 866 IBD genes are out of the 4477 differentially expressed genes (DEGs) we identified from gene expression analysis. Let us name these 318 genes as IBD related differentially expressed genes (IDEGs) and the rest 4159 as only differentially expressed genes (ODEGs). In this work we consider these 318 genes as known IBD genes.

**Fig. 3:**
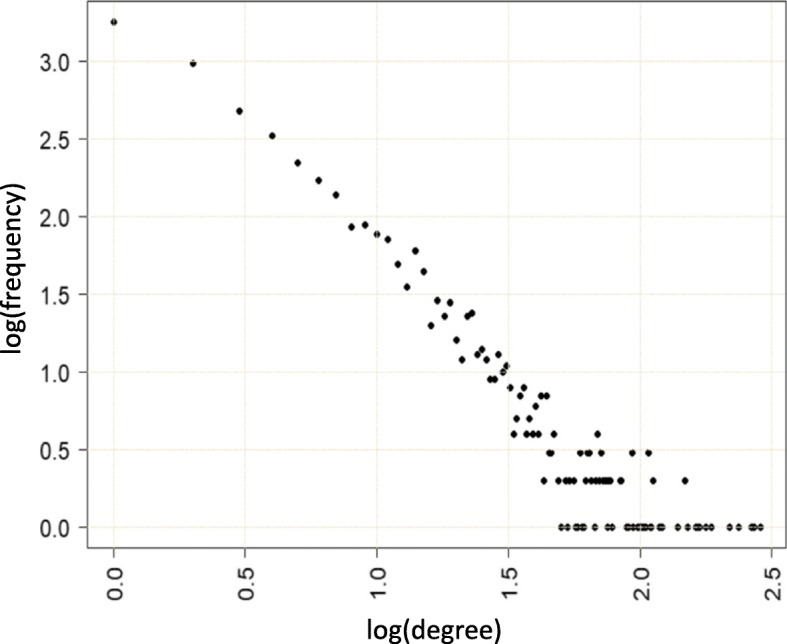
Degree distribution of the IBD related PPI network follows the power law

We constructed a disease related PPI network based on Human Integrated Protein-Protein Interaction rEference (HIPPE) database [27]. In HIPPE database each interaction is reported with a confidence score. We first extracted the interactions involving ODEGs with a score greater than 0.7, which included 4135 ODEGs. We then retrieved the interactions involving all 318 IDEGs with a score greater than 0.1. Thus we retrieved a total of 38,500 interactions involving IDEGs, ODEGs and other genes (OGs). From these interactions, we empirically selected interactions to construct the final PPI network according to following criterion: IDEG-IDEG:0.1, IDEG-ODEG:0.1, IDEG-OG:0.72, ODEG-IDEG:0.1, ODEG-ODEG:0.1, ODEG-OG:0.85. In summary, we gave the highest priority to interactions involving IDEGs (genes that are both known IBD genes and differentially expressed genes according to the expression data we used). Also, most priority was given to interactions for which both genes are ODEGs (only differentially expressed genes). These genes are likely to contain system level information of molecular mechanism of IBD. The HIPPIE database recommend 0.72 as a good score which we used for IDEG-OG interactions and finally adjusted 0.85 for ODEG-OG interactions to roughly keep similar number of DEGs (IDEGs + ODEGs) and OGs (Other Genes) in the PPI network for the sake of balance and thus extracted unbiased information. Finally we selected 16,429 interactions involving 5056 genes with 291 IDEGs, 2072 ODEGs and 2693 OGs. The degree distribution of the network is shown in Fig. 3. As many other typical PPI networks, the degree distribution of our constructed network followed power law. Some other global network properties of the network include average path length 4.18, clustering co-efficient 0.1 and diameter 11. For such a big network the clustering coefficient of 0.1 is substantialy enough indicating presence of densely connected clusters in the network.

### Clustering of the PPI network

After creating the disease related PPI network we determined clusters in the network by DPClusO algorithm. DPClusO generates overlapping clusters and ensures coverage. For example, each node goes to at least one cluster. We hypothesize that clustering of a disease relevant PPI network helps isolate systems with disease related properties and therefore statistically significant clusters enriched with known IBD genes can be used to predict novel IBD genes and pathways based on the associations determined by combined information of IBD gene expression and protein-protein interactions.

We generated 9 sets of clusters from the PPI network by DPClusO algorithm using density values of 0.1, 0.2, 03, 0.4, 0.5, 0.6, 0.7, 0.8 and 0.9. Table 1 shows characteristics, i.e. the number of clusters, size of the biggest cluster and average cluster size, related to the clusters generated by the 9 different density values. As expected, smaller density value resulted in larger and fewer number of clusters generated. To assess the enrichment of IDEGs in each of the identified clusters we determined Fisher’s exact test *p*-values. In this work we proposed to consider statistically significant clusters for predicting novel IBD related genes and pathways. Therefore we assigned a score called SScore (Significance Score) to each gene as a measure of confidence of prediction based on the *p*-values of the clusters they belong to. The definition of SScore is provided in the Methods section. Based on these scores we performed ROC analysis to determine which set of clusters should be used for predicting novel IBD genes.

**Table 1:** Characteristices of the clusters generated with different input densties using the DPClusO algorithm based on the IBD related PPI network

density	#ofcluster	maxsize	avgsize
0.1	827	70	18.52
0.2	1229	42	10.69
0.3	1779	31	7.18
0.4	2219	21	5.74
0.5	2790	16	4.61
0.6	3597	13	3.53
0.7	4425	11	2.59
0.8	4534	9	2.50
0.9	4775	7	2.31

### ROC analysis

**Fig. 4:**
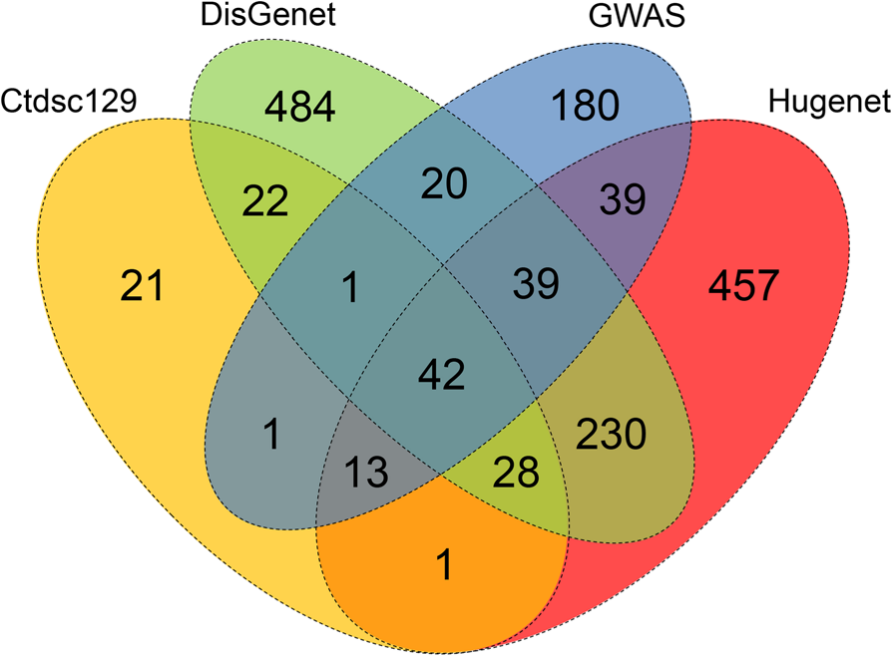
Venn diagram showing overlapping between IBD genes collected from four different sources

**Fig. 5:**
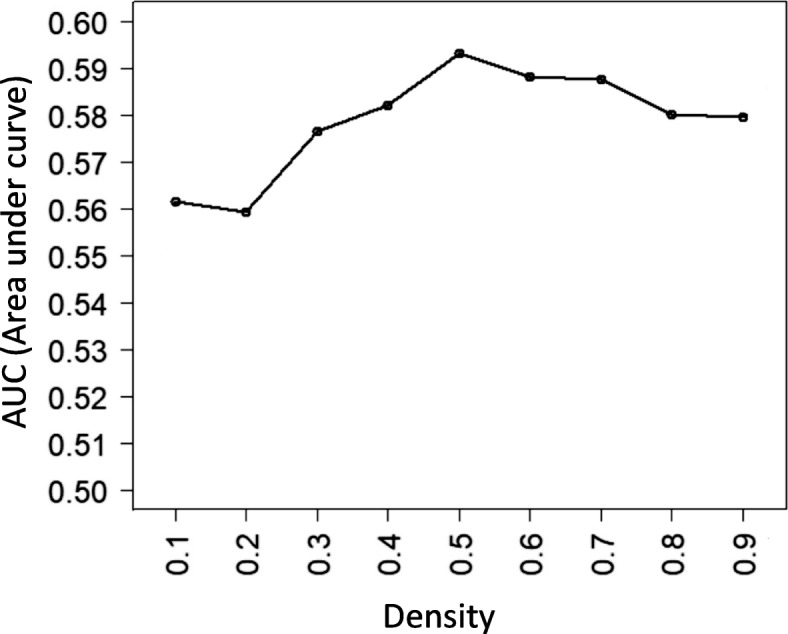
AUCs corresponding to 9 sets of clusters

In our disease relevant PPI network there are total 5056 genes out of which 291 genes are IDEGs which are among the 318 genes considered as known IBD genes in the present work. We predicted the degree of relevance of the rest 4765 genes with IBD based on SScore. We collected well curated and well studied IBD genes from 3 databases as follows, The Comparative Toxicogenomics Database (CTD) [28], DisGeNet [26], HuGENet [29] and published literatures on results of GWAS [30–33]. The venn diagram of the reported IBD genes in these 4 databases is shown in Fig. 4. It is noticeable that IBD genes listed by these 4 sources are substantially different, indicating the need for finding comprehensive set of potential IBD genes. Although these four sources are not the complete list of IBD genes, they can be used to assess the effectiveness of SScore. The ROC curves corresponding to the 9 sets of clusters are very similar, which imply the underlying signal in the carefully constructed PPI network is very strong and DPClusO algorithm has been successful to catch the signal for across a wide range of the input parameter. Figure 5 shows the Area Under the Curve (AUC) for the 9 ROC curves. The AUCs are not very high, which may be due to incomplete information of known good quality IBD genes. We observed that the highest AUC was obtained in the case of the cluster set generated using density =0.5. So we selected the genes included in the statistically significant clusters of this set, adjusted the corresponding *p*-values for multiple testing [34] and selected the genes having adjusted *p*-values less than 0.05 as predicted IBD genes.

### Prediction and validation

We predicted 909 genes (with adjusted *p*−*value*<0.05) included in the clusters selected from the set corresponding to the highest AUC as our predicted IBD genes. These 909 genes are other than the genes considered as known IBD genes (IDEGs) in this work. The list of the 909 predicted IBD genes and corresponding adjusted *p*-values are shown in Additional file 1. To validate our results we initially searched how many of the predicted genes are exactly matched with well curated known IBD genes. We found 83, 8, 54, 22 of the predicted genes matched with reported IBD genes in (1) HuGeNet, (2) CTD, (3) DisGeNet databases and (4) GWAS results respectively. After considering overlapping between databases, 14.5% of our predicted genes matched with good quality known IBD genes. Given the fact that we made predictions based only on a specific gene expression data and a limited set of known IBD genes, the 14.5% matching with good quality data is significant (*p*−*value*<3.45×10^−12^, *p*-value determined based on hypergeometric ditsribution assuming total number of human genes as 20000). However, our approach is a computational approach. So, it is rational to compare our result also with computationally predicted IBD genes. In CTD database other than the good quality curated set there is a big set of genes inferred as IBD genes by various methods. When we compare our result with this big set, we find that 93.8% of the genes we predicted matched with reported IBD genes (*p*−*value*<9.8×10^−14^). As we have predicted the genes by wisely integrating the information of gene expression and protein-protein interaction, it is very likely that they are truely related to IBD. One of the predicted genes IL12B is supported by all four above-mentioned sources as an IBD related gene. IL12B and IL23R have been identified as susceptibility genes for IBD by recent genome-wide association studies [35]. Each of the three genes CCR5, IL1R2 and LTA is mentioned as IBD related gene in three of the above mentioned sources. High expression of CCR5 has been reported in active IBD [36]. Epithelial IL1R2 takes part in homeostatic regulation during remission of ulcerative colitis [37]. It has been reported that LTA elicits a strong inflammatory reaction controlled by intestinal dendritic cells [38]. Thus we have found IBD relevance of many other predicted genes by literature review. The proposed method, however is a computational one and the role of the newly predicted genes in IBD pathogenesis should be clarified by further studies.

The degree of relevance of the 909 genes (shown in Additional file 1) predicted by the proposed approach can be evaluated by the corresponding *p*-values. The top 20 predicted novel IBD genes (not reported in any of the four sources of Fig. 4) based on *p*-values are IKBKG, BIRC3, BCL10, RNF31, RBCK1, CCRL1, LAMC3, CARD11, KISS1, THBS2, TRAF2, TRAF1, PYCARD, MIS12, ALB, AR, RIPK1, SHARPIN, SNAPIN and ITGA2B. Many of these 20 top IBD risk genes we identified from this study have been found to be associated with IBD. In human, the IKBKG gene encodes NF- *κ*B essential modulator (NEMO) which is an inhibitor of nuclear factor *κ*B kinase subunit gamma (IKK- *γ*) [39]. NEMO (IKK- *γ*) is the regulatory subunit of the inhibitor of the I- *κ*B kinase (IKK) complex, that activates NF- *κ*B causing activation of genes involved in inflammation, immunity, cell survival, and other pathways. IBD-like immunopathology can be developed by IKBKG [40]. BIRC2 and BIRC3 are important genes in regulating the expression of proinflammatory cytokines, such as TNF- *α*, through NF- *κ*B and MAPK pathways [41]. BCL10 is an adaptor protein which is assumed to play role in the PAF-induced inflammatory pathway in human intestinal epithelial cells [42]. RNF31 and HOIL-1L complex functions in linear ubiquitination of proteins in the NF- *κ*B pathway in response to proinflammatory cytokines [43]. CCRL1 acts as a functional receptor for the monocyte chemoattractant protein family of chemokines; elevated chemokine expression is associated with many inflammatory diseases such as IBD, rheumatoid arthritis and asthma [44, 45]. As a component of the LUBAC complex, RBCK1 conjugates linear (Met1-linked) polyubiquitin chains to substrates and thus plays imoportant role in NF- *κ*B activation and inflammation regulation [46]. RBCK1-deficiency is associated with autoinflammatory syndrome and immunodeficiency [46]. LAMC3 is expressed saliently at significantly different proportions in low and high coherence expression profiles of IBD patients [47]. The elevated stromal protein thrombospondin-2 (THBS2) has been reported to be a part of a fibroblast-specific inflammation signature [48]. It has been shown that TRAFs are important mediators of innate immune receptor signaling [49]. IBD and IBD recurrence is associated with the overexpression of TRAF2 [50–52]. TRFA1 is reported to be highly expressed in IBD patients [53]. To form the basic Inflammasome subunit, the adaptor protein ASC (encoded by the PYCARD gene) links the NLR sensor to caspase-1 [54]. TNF- *α*-induced necroptosis is associated with two members of the receptor-interacting protein (RIP) family of kinases – RIPK1 and RIPK3 [55]. Tumor necrosis factor- *α* (TNF- *α*) can bind to one of two receptors, TNFR1 or TNFR2; TNFR activation results in the activation of NF- *κ*B leading to the induction of proinflammatory cytokines [55].

### Comparison with ToppGene

It has been demonstrated that ToppGene [56] performs better than several other methods such as SUSPECTS [57], PROSPECTOR [58], ENDEAVOUR [59] in candidate gene prioritization. From the ToppGene suite [60] we used ToppGenet which is a web based tool that can take input a set of seed genes and can return a list of genes with closely related roles with a prioritization score. In our work, based on gene expression data and DisGeNet database we considered 318 genes as known IBD genes and based on those we predicted 909 other genes as IBD related genes. We assigned the same 318 genes to ToppGenet and from the output we selected the highest ranking 909 genes which we compared with the 909 genes determined by our approach. For both sets, we determined the number of genes matched with the union of reported IBD genes in 4 sources of Fig. 4. Also we determined the AUCs using prioritization score and SScore in case of ToppGenet and our approach respectively. In case of ToppGenet, we selected network based approach as our approach is also network based. Furthermore, we used 3 available options for ToppGenet as follows: (i) K-Step Markov, (ii) Page rank with priors and (iii) Hits with priors. The comparison results are shown in Table 2. The results show that performance of our approach is comparable in terms of the number of identified genes and better in terms of AUC.

**Table 2:** Results of comparison with ToppGene

Parameter of comparison	ToppGenet			Our Method
	K-step Markov	Page rank with priors	Hits with priors	
Number of match	163 >	169 >	102 <	132
AUC	0.4969 <	0.4339 <	0.4831 <	0.5826

### Gene ontology and pathway analysis

As a group the top 20 predicted genes (names mentioned in the previous section) are enriched in some important BP(Biological Process) related GO terms, such as I- *κ*B kinase/NF- *κ*B signaling, positive regulation of immune response, regulation of tumor necrosis factor-mediated signaling pathway and MF(Molecular Function) terms, such as ubiquitin protein ligase binding, identical protein binding. We also performed enrichment analysis for all of the 909 genes. Some significant BP related GO terms enriched in these genes are nitrogen compound metabolic process, response to stimulus, immune system process, cell surface receptor signaling pathway, response to stress, response to lipid, positive regulation of leukocyte cell-cell adhesion and MF terms are enzyme regulator activity, kinase activity, protein complex binding, histone deacetylase binding, transcription factor activity, protein binding, protein C-terminus binding. NF- *κ*B pathway mediate events including the activation of genes encoding inflammatory molecules and is found to be chronically active in IBD [61]. All the above mentioned GO terms associated to a group of genes were searched by using the enrichment analysis tool [62] provided in the web page of Gene Ontology Consortium.

**Fig. 6:**
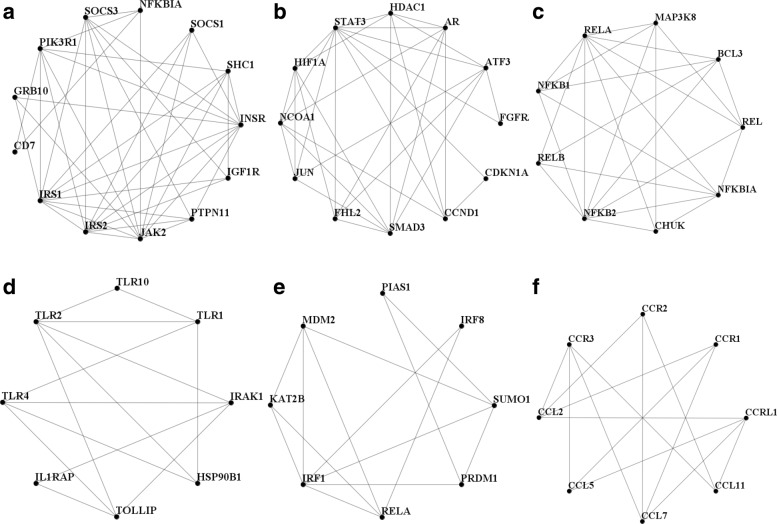
(**a**)-(**f**) Examples of statistically significant clusters

As examples we arbitrarily select and show 6 of the statistically significant clusters in Fig. 6(a)-(f). In these clusters 4, 5, 4, 4, 3, 5 genes are IDEGs respectively and 3, 2, 3, 2, 2, 2 genes are reported to be IBD genes by 4 reliable sources as mentioned in Fig. 4. Many of the genes included in these clusters are related to IBD. It has been reported that SOCS deficient mice develop severe colitis (similar to human ulcerative colitis) depending on some factors [63]. Expression of IGF1R in submucosal fibroblast-like cells, subserosal adipocytes and hypertrophic plexus has been confirmed to be CD specific, indicating relations between IGF1R and chronic inflammation [64]. It has been reported that the deficit of PTPN11 is related to the severity of colitis [65]. IRF8 promotes the production of IL12 and IL23 in the development of experimental autoimmune encephalomyelitis and inhibits the production of IL27, and thus forms a cytokine environment suitable for differentiation and maintenance of Th1 cells and Th17 cells and also, IRF8 exacerbates inflammation by activating microglia [66]. C-C motif chemokine receptors, CCR1 and CCR3 are membrane proteins that particulaly bind and respond to cytokines of the CC chemokine family [67, 68].

Based on significant *p*-values, we empirically selected some enriched BP and MF terms for these clusters. Some important BP related GO terms enriched in these clusters (a)-(f) are as follows: (a) cell surface receptor signaling pathway, regulation of cellular response to insulin stimulus, cellular response to hormone stimulus, (b) negative regulation of programmed cell death, response to endogenous stimulus, cell differentiation, (c) regulation of cytokine production, intracellular signal transduction, regulation of type I interferon production, (d) toll-like receptor signaling pathway, activation of innate immune response, inflammatory response, (e) regulation of transcription from RNA polymerase II promoter, negative regulation of transcription, DNA-templated, negative regulation of nitrogen compound metabolic process, (f) chemotaxis, inflammatory response, positive regulation of MAPK cascade and MF related GO terms are as follows: (a) phosphatidylinositol 3-kinase binding, insulin receptor binding, receptor binding (b) transcription factor binding, regulatory region DNA binding, chromatin binding, (c) transcription factor activity, sequence-specific DNA binding, chromatin binding, (d) signal transducer activity, Toll-like receptor binding, (e) SUMO transferase activity, ubiquitin-like protein ligase binding, (f) G-protein coupled receptor binding, cytokine receptor activity.

**Fig. 7:**
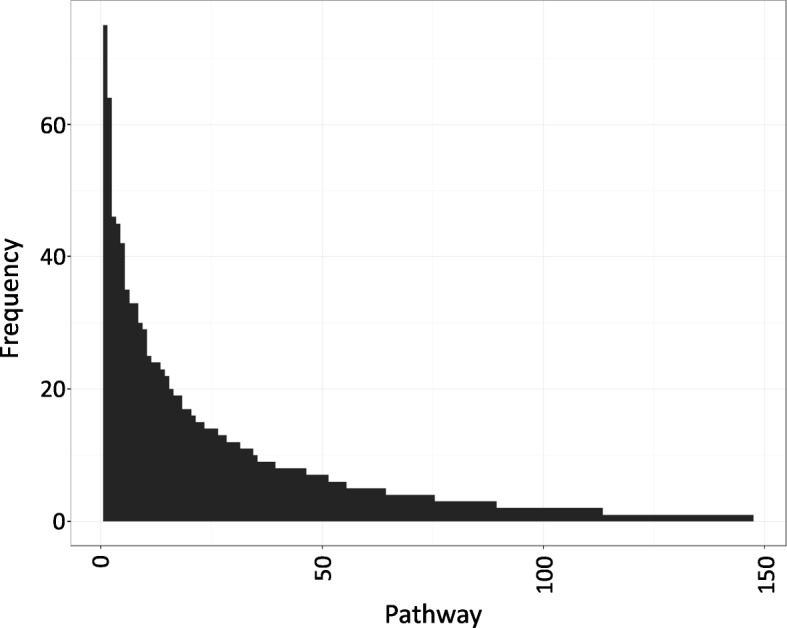
Frequencies of pathways related to statistically significant clusters

We hypothesize that clustering disease related PPI network helps isolate systems with disease related properties. Therefore, we selected 442 statistically significant clusters (*p*−*value*<0.05). We use these statistically significant clusters to determine IBD related pathways. We separately mappled the genes included in each of the statistically significant clusters to KEGG pathway [69]. For each cluster we determined the top three pathways based on the association of majority number of genes. Additional file 2 shows the selected pathways and enriched GO terms for these clusters. Frequencies of these selected pathways are shown in histograms of Fig. 7. The top 10 pathways with the highest frequency are : (1) MAPK signaling pathway, (2) Chemokine signaling pathway, (3) Cytokine-cytokine receptor interaction, (4) Pathways in cancer, (5) Toll-like receptor signaling pathway, (6) Cell cycle, (7) NOD-like receptor signaling pathway, (8) Apoptosis, (9) Endocytosis, (10) Focal adhesion. Particularly interested pathways associated with IBD in these results are MAPK, Chemokines, Cytokines, Toll-like receptors, and NOD-like receptor pathway. Previous studies have shown that these predicted pathways are highly relevant to IBD.

MAPK signaling pathway are evolutionarily conserved kinase modules whose fanctions are to transmit extracellular signals to various machinery inside the cell that manage fundamental cellular processes such as growth, differentiation, migration, proliferation and apoptosis. Activation of ERK1/2 by growth factors depends on the MAPKKK c-Raf, but other MAPKKKs may activate ERK1/2 in response to pro-inflammatory stimuli [70]. Small chemoattractant peptides called Chemokines provide directional cues for the cell trafficking and therefore are important for protective host response. They are soluble factors which play key roles in regulating immune cell recruitment during inflammatory responses and defense againsst foreign pathogens. Soluble extracellular proteins or glycoproteins known as Cytokines are crucial intercellular regulators and mobilizers of cells involved in inherent as well as adaptive inflammatory host defenses, cell death, cell growth, angiogenesis, differentiation and development and repair processes targeting the restoration of homeostasis. It has been reported that cytokines/chemokines are engaged in not only the initiation but also the persistence of pathologic pain by activating nociceptive sensory neurons. There are inflammatory cytokines engaged in nerve-injury/inflammation-induced central sensitization, and are associated to the development of contralateral hyperalgesia/allodynia [71, 72]. Toll-like receptors (TLRs) are a family of pattern recognition receptors that are best-known for their role in host defence from infection. It has been reported that TLRs play important role in maintaining tissue homeostasis by regulating the inflammatory responses to injury [73]. The intracellular NOD-like receptor (NLR) family contains more than 20 members in mammals and plays a pivotal role in the recognition of intracellular ligands. The activated state of caspase-1 regulates maturation of the pro-inflammatory cytokines IL-1B, IL-18 and drives pyroptosis [74].

## Conclusions

We presented a method for predicting IBD related genes and pathways by integrating the information of IBD gene expression and protein-protein interactions and a set of known IBD genes from DisGeNet database. We determined differentially expressed genes (DEGs) based on IBD gene expression data and constructed a IBD relevant PPI network using DEGs and known IBD genes. We extracted high density modules from the PPI network using our graph clustering algorithm DPClusO. We determined modules enrichment with known IBD genes by Fisher’s exact test and used those statistically significant modules to predict novel IBD genes and pathways. We compared our results with several other databases and published literatures. We found 93.8% of our predictions are found in these published results. Specially we found our results substantially matched with IBD genes collected in curated databases and high-profile publications.

Furthermore, based on our ranking score, we selected top 20 predicted novel IBD genes and by literature survey we observe that most of these genes are really substantially related to IBD. As a group these 20 genes are enriched in the GO term I- *κ*B kinase/NF- *κ*B signaling. NF- *κ*B pathway mediates events including the activation of genes encoding inflammatory molecules and is found to be chronically active in IBD. Also, based on statistically significant clusters we identified top 10 IBD related pathways which include MAPK signaling pathway, Chemokine signaling pathway, Cytokine-cytokine receptor interaction etc. These pathways play roles in inflammation related diseases including IBD.

Finding disease-causal genes is the part of the process to understand disease mechanism and develop drugs that can provide synergistic effects targeting many genes/proteins simultaneously. This study discussed a computational approach to reach these goals in the context of IBD. The proposed method can also be applied to find disease-causal genes related to other diseases.

## Methods

### Data collection and preprocessing

We downloaded the IBD gene expression data from NCBI’s Gene Expression Omnibus (GSE57945) [25]. The gene expression data was generated using TopHat [75]. The samples were collected for three biological groups: healthy control, Crohn disease and ulcerative colitis [24]. We removed genes with expression values equaling to zero across all samples. The final expression data set included 14664 genes and 322 samples, which included 42 control samples, 218 CD samples, and 62 UC samples. We also downloaded reported IBD genes from several other databases, such as The Comparative Toxicogenomics Database (CTD) [28], DisGeNet [26], HuGENet [29]. The protein-protein interaction data was downloaded from HIPPE database [27].

### Identifying differentially expressed genes

We performed differential expression analysis using the R package edgeR, which is based on negative binomial models [76]. We implemented the exact test for a difference in mean between two groups of negative binomial random variables by using edgeR after applying Trimmed Mean of M-value(TMM) normalization [77, 78] to data. False discovery rate (FDR) was estimated from unadjusted *p*-values using Benjamini Hochberg multiple testing method [34, 79].

### Network clustering by DPClusO

DPClusO is a graph clustering algorithm [80], which is the updated version of DPClus algorithm [81]. DPClusO can extract densely connected nodes in a network as a cluster or module. Particularly, it produces overlapping clusters or modules since genes can be disease-causal genes in multiple diseases or have multiple biological functions and are involved in multiple pathways. This algorithm can be applied to an undirected graph *G*=(*N*,*E*) that consists of a finite set of nodes *N* and a finite set of edges *E*. Two important parameters used in this algorithm are density *d*_*k*_ and cluster property *cp*_*nk*_. Density *d*_*k*_ of cluster *k* is the ratio of the number of edges present in the cluster (|*E*|) and the maximum possible number of edges in the cluster (|*E*|_*max*_). The cluster property *cp*_*nk*_ of node n with respect to cluster *k* is expressed by the follow equation:

$$\begin{array}{@{}rcl@{}} {cp}_{nk}&=&\frac{E_{nk}}{{d_{k}}\times{N_{k}}} \end{array} $$

*N*_*k*_ is the number of nodes in cluster *k*. *E*_*nk*_ is the total number of edges between the node *n* and each of the nodes of cluster *k*.

### Fisher’s exact test

We evaluated the enrichment of the known IBD genes (referred to as IDEGs in the present work) in the clusters from our PPI analysis using Fisher’s exact test. The test is an alternative statistical significance test used in the analysis of 2×2 contingency tables [82, 83].

To do this, for each cluster we determined the values of *a*, *b*, *c*, and *d* as demonstrated in the following table:

**Table:** 

	IBD Genes	Non-IBD Genes	
In Cluster	*a*	*b*	*a*+*b*
Not in Cluster	*c*	*d*	*c*+*d*
	*a*+*c*	*b*+*d*	*n*

Here *n* is the total number of genes in the network.

### SScore

We assigned a score called SScore (Significance Score) to each gene as a measure of confidence of prediction based on the *p*-values of the clusters they belong to. By definition *SScore*=−*log*(*p*−*value*). As DPClusO generates overlapping clusters, a gene may belong to more than one clusters and thus may correspond to more than one *p*-values. We used the lowest *p*-value corresponding to a gene to calculate its SScore.

### ROC Analysis

We evaluated the power of SScore to predict the known IBD genes by performing receiver operating characteristic (ROC) analysis [84, 85]. The ROC curve was created by selecting a series of threshold SScore values to generate True Positive Rate (TPR) and False Positive Rate (FPR). TPR is the proportion of true positive predictions out of all the positive data and FPR is the proportion of false positidve predictions out of all the negative data and can be expressed by the following equations:

$$\begin{array}{@{}rcl@{}} TPR=\frac{TP}{TP+FN} \;\;\;\;\;\;\;\; FPR=\frac{FP}{FP+TN} \end{array} $$

Corresponding to a certain threshold SScore th, false positive (FP), true positive (TP), false negative (FN) and true negative (TN) are defined as follows: TP is the number of reported IBD genes having *SScore*≥*th*, FP is the number of non-IBD genes having *SScore*≥*th*, TN is the number of non-IBD genes having *SScore*<*th*, and FN is the number of reported IBD genes having *SScore*<*th*.

We observed the performance of SScore to identify known IBD genes by using the Area Under the ROC Curve (AUC) analysis [86]. In term of AUC analysis, we used R package named ROCR [87]. We considered a prediction as ’True’ prediction if a gene is reported as IBD gene in any of the following four sources: (1) Human Genome Epidemiology Network (HuGENet), (2) Comparative Toxicogenomics Database (CTD), 3) DisGeNet database and (4) GWAS results [30–33]. Here, FP, TP, FN, TN were calculated based on known information i.e. without having knowledge of all IBD related and unrelated genes. Therefore, the calculated TPR and FPR values were affected by the unknown nature of the TN and FN genes.

## Additional files

Additional file 1 List of predicted IBD genes. (XLSX 26 kb)

Additional file 2 Significant clusters with selected pathways and enriched GO terms associated to them. (XLSX 31 kb)

## Abbreviations

AUC: Area under the curve
BP: Biological process
CD: Crohn’s disease
CTD: The comparative toxicogenomics database
DEG: Differentially expressed gene
GO: Gene ontology
GWAS: Genome-wide association studies
HIPPIE: Human integrated protein-protein interaction rEference
IBD: Inflammatory bowel disease
IDEG: IBD related differentially expressed gene
MF: Molecular function ODEG: Only differentially expressed gene
OG: Other gene
PPI: Protein-protein interaction
SNP: Single nucleotide polymorphisms
UC: Ulcerative colitis
